# Understanding Mobile Health and Youth Mental Health: Scoping Review

**DOI:** 10.2196/44951

**Published:** 2023-06-16

**Authors:** Xiaoxu Ding, Kelli Wuerth, Brodie Sakakibara, Julia Schmidt, Natalie Parde, Liisa Holsti, Skye Barbic

**Affiliations:** 1 Faculty of Medicine Department of Occupational Science and Occupational Therapy University of British Columbia Vancouver, BC Canada; 2 Faculty of Graduate Studies, Rehabilitation Science, University of British Columbia Vancouver, BC Canada; 3 Foundry, Providence Health Care Vancouver, BC Canada; 4 Faculty of Medicine, Centre for Chronic Disease Prevention and Management, University of British Columbia Kelowna, BC Canada; 5 Department of Computer Science, University of Illinois Chicago Chicago, IL United States; 6 Natural Language Processing Laboratory, University of Illinois Chicago Chicago, IL United States; 7 Centre for Health Evaluation & Outcome Sciences Vancouver, BC Canada

**Keywords:** adolescent, COVID-19, engagement, health outcome, illness, implementation, mental disorder, mental health, mHealth intervention, mHealth tools, mHealth, policy, scoping review, young adult, youth

## Abstract

**Background:**

A total of 75% of people with mental health disorders have an onset of illness between the ages of 12 and 24 years. Many in this age group report substantial obstacles to receiving quality youth-centered mental health care services. With the rapid development of technology and the recent COVID-19 pandemic, mobile health (mHealth) has presented new opportunities for youth mental health research, practice, and policy.

**Objective:**

The research objectives were to (1) synthesize the current evidence supporting mHealth interventions for youths who experience mental health challenges and (2) identify current gaps in the mHealth field related to youth’s access to mental health services and health outcomes.

**Methods:**

Guided by the methods of Arksey and O’Malley, we conducted a scoping review of peer-reviewed studies that used mHealth tools to improve youth mental health (January 2016-February 2022). We searched MEDLINE, PubMed, PsycINFO, and Embase databases using the following key terms: (1) mHealth; (2) youth and young adults; and (3) mental health. The current gaps were analyzed using content analysis.

**Results:**

The search produced 4270 records, of which 151 met inclusion criteria. Included articles highlight the comprehensive aspects of youth mHealth intervention resource allocation for targeted conditions, mHealth delivery methods, measurement tools, evaluation of mHealth intervention, and youth engagement. The median age for participants in all studies is 17 (IQR 14-21) years. Only 3 (2%) studies involved participants who reported their sex or gender outside of the binary option. Many studies (68/151, 45%) were published after the onset of the COVID-19 outbreak. Study types and designs varied, with 60 (40%) identified as randomized controlled trials. Notably, 143 out of 151 (95%) studies came from developed countries, suggesting an evidence shortfall on the feasibility of implementing mHealth services in lower-resourced settings. Additionally, the results highlight concerns related to inadequate resources devoted to self-harm and substance uses, weak study design, expert engagement, and the variety of outcome measures selected to capture impact or changes over time. There is also a lack of standardized regulations and guidelines for researching mHealth technologies for youths and the use of non–youth-centered approaches to implementing results.

**Conclusions:**

This study may be used to inform future work as well as the development of youth-centered mHealth tools that can be implemented and sustained over time for diverse types of youths. Implementation science research that prioritizes youths’ engagement is needed to advance the current understanding of mHealth implementation. Moreover, core outcome sets may support a youth-centered measurement strategy to capture outcomes in a systematic way that prioritizes equity, diversity, inclusion, and robust measurement science. Finally, this study suggests that future practice and policy research are needed to ensure the risk of mHealth is minimized and that this innovative health care service is meeting the emerging needs of youths over time.

## Introduction

### Mental Illness

Mental health disorders are the leading cause of disability worldwide and are considered a global public health challenge [1,2]. Globally, 1 in 4 people are affected by mental health disorders each year; up to 50% of people experience mental health challenges in their lifetime [3]. The global burden of disease (GBD) is compounded by the “mental health treatment gap,” referring to those people in need of mental health treatment but who have not received it. People with mental health challenges experience different levels of barriers to accessing specialized care when needed [4]. Such barriers are multifaceted and may include poor mental health literacy, social stigma, trust and confidentiality issues with health professionals, and systemic difficulties such as financial hardship [5].

A total of 75% of people with mental health disorders have an onset of illness between the ages of 12 and 24 years [6]. This is a peak period of development for youths (defined here as ages 12-24 years); it is often the life stage to pursue education or begin a career, to build social relationships, and to explore new interests [7]. Yet, youths experience the worst levels of access to mental health care from poorly designed, grossly underresourced, and typically unfriendly health care services [8]. Current research indicates the need for a full range of interventions for youths [9], including health promotion [10,11], early intervention [12], and long-term supports such as integrated self-management [13], community outreach [14], and hospital care [15]. With existing barriers compounded by the COVID-19 pandemic, in-person mental health care is more challenging than ever to navigate and access for youths [16]. In response, mobile health (mHealth), a term used to describe the collective set of digital mental health interventions, has been proposed as a solution to meet the needs of youths across the world.

### mHealth

In 2022, mobile phone users reached 6.5 billion worldwide, accounting for approximately 80% of the global population. In developed countries such as Canada and the United States, 97% of people own a mobile phone and 85% use a smart phone [17,18]. Such mass usage is motivating the rapid growth of medical software and apps for diverse populations, including youths [19]. These mobile device–based apps cover multiple domains of technologies used to assess, capture, or support areas of health, including physical activity and fitness, diet, emotional and mental health, and health services. mHealth technologies have been used to track vital body signs, such as blood pressure, heart rate, exercise, sleep activity, nutritional values in meals, mental health and wellness, anxiety, and mood [20]. A total of 54,603 health care and medical apps were available on the Google Play Store in August 2022, up by almost 4% compared to the previous quarter [21].

While the pace of new mHealth technologies is growing rapidly, the regulations and standards for their use have not yet followed. In a recent study of nearly 300 apps for mental health, less than one-third received input from a mental health expert [22]. There is also little consensus regarding standards for mHealth tools, not to mention the effectiveness of mHealth interventions on diverse populations, including youths experiencing mental health challenges.

In summary, the field of mHealth often presents a dichotomy of opportunity and risk. On the one hand, the lack of standards and regulation for mHealth apps presents global concerns for the safety of youths, notably personal security and the uptake of misinformation [23-25]. On the other hand, mHealth may have an increasingly important role in health promotion, education, and interventions to bridge the gap for those who cannot access in-person services [26]. A greater understanding is needed to learn about how youths use mHealth technologies in their daily lives, with specific emphasis on understanding how they navigate safety risks and use these technologies to improve health and wellness outcomes.

The purpose of this scoping review is to synthesize the current evidence supporting mHealth interventions for youths accessing support for mental health challenges. This will facilitate understanding of what is missing in the field of mHealth and support recommendations for research, practice, and policy. The specific objectives are to (1) synthesize the current evidence supporting mHealth interventions for youths who experience mental health challenges and (2) identify current gaps in the mHealth field, with an overarching goal of improving youths’ mental health service access, outcomes, and experiences.

## Methods

### Overview

We conducted a scoping review to examine the extent, range, and nature of mHealth and to identify gaps in the existing literature on this emerging topic. This scoping study followed the 5 stages of Arksey and O’Malley’s scoping study framework [27] to (1) identify the research question; (2) identify relevant studies; (3) select studies; (4) chart the data; and (5) collate, summarize, and report the results.

### Step 1: Identify the Research Question

What is known in the existing literature about the feasibility and effectiveness of mHealth intervention for youths, aged 12-24 years, facing mental health challenges?

### Step 2: Identify Relevant Studies

Under the guidance of a medical librarian, a comprehensive search of the following electronic databases was conducted: MEDLINE (Ovid), Embase (Ovid), PsycINFO (EBSCO), and PubMed (see Multimedia Appendix 1 for example search). We consulted with mHealth stakeholders and youths to decide on the range of dates to search. Our expert team highlighted rapid changes in the field, notably the influence of TikTok after its launch in 2016. To ensure relevance and reference value, we decided to only review articles published during the past 6 years (January 1, 2016, to February 7, 2022) to manage the scope, breadth, and rapidly changing information available. Key terms derived from the research question were selected and expanded to create a comprehensive list of search terms, including “telemedicine,” “telerehabilitation,” “mobile applications,” “mHealth (mobile health),” “eHealth (electronic/digital health),” and “telehealth,” as well as a combination of the following mental health condition-related terms: “mental disorders,” “anxiety,” “depression,” “eating disorder,” “schizophrenia,” “bipolar,” “obsessive compulsive disorder,” and “posttraumatic stress disorder,” along with a list of key terms to define the age group of this review: “adolescent,” “teen,” “youth,” and “young adult.” Combinations of these terms, along with Medical Subject Heading (MeSH) terms, were tested iteratively in each of the databases selected to inform the new combination of different terms leading to relevant literature. All searches included at least one identifier for mHealth (eg, telehealth and eHealth), 1 identifier for mental health condition (eg, depression and anxiety), and 1 identifier for age range (eg, youth and young adult). The lead author reviewed the title and abstract of each study to determine eligibility based on predetermined inclusion and exclusion criteria (described below) after duplicates were removed. After the completion of the initial review, the articles were thoroughly reviewed by the lead author based on the research topic.

### Step 3: Select Studies

The following inclusion criteria were considered: (1) published in English; (2) published between January 1, 2016, and February 7, 2022; (3) included human subjects, whose ages fall between 12 and 24 years; (4) included at least one mHealth intervention tool targeting 1 or more mental health conditions for youths; and (5) referenced literature from peer-reviewed journals and book chapters. Exclusion criteria were as follows: (1) editorial comments, commentaries, book reviews, and opinion articles; (2) incomplete studies (eg, description of intervention, protocols, ideas from symposia, and conference summaries); (3) articles without full text available. Relevant systematic reviews were included in the study to serve as background literature but were excluded from the data extraction and analysis process to focus on intervention studies.

### Step 4: Chart the Data

Through careful review of the literature, the researchers (XD and SB) identified the key components and issues discussed in all relevant studies. This information was recorded in a data extraction sheet, along with information on each study (author, publication year, population demographics, location, study design, level of evidence, characteristics of the intervention, targeted health condition, and outcomes).

### Step 5: Collate, Summarize, and Report Results

The information was synthesized and used to map out the scope and breadth of included literature on the topic of mHealth intervention for youths’ mental health challenges.

## Results

### Overview

**Figure 1:**
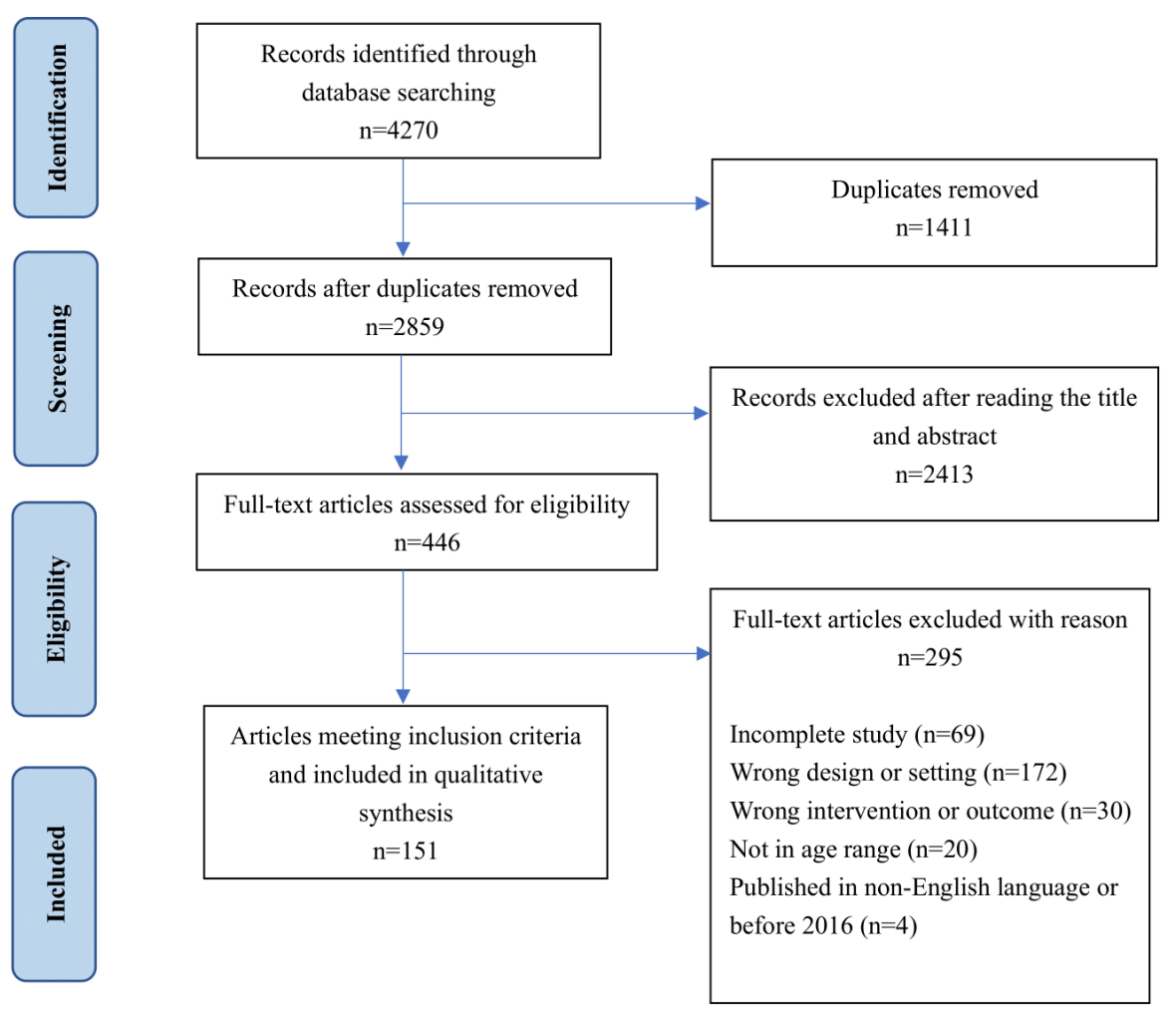
PRISMA (Preferred Reporting Items for Systematic Reviews and Meta-Analyses) diagram describing the search process and the number of articles meeting inclusion criteria for this study (N=151).

As noted in Figure 1, the search identified 4270 citations for initial screening. As noted in Figure 1, a total of 1411 duplicates were removed, resulting in 2859 citations for title and abstract reviews. A further 2413 articles were excluded because their title or abstract did not address mHealth interventions for youths’ mental health, leaving 446 citations. After full text review, a total of 151 articles met the inclusion criteria for this study. Table 1 summarizes the countries of origin of the included studies.

**Table 1:** Countries of origin of papers included in the scoping review.

Country	Articles (N=151), n %
United States	61 (40)
Australia	28 (19)
Canada	10 (7)
Sweden	10 (7)
United Kingdom	8 (6)
New Zealand	7 (5)
China	4 (3)
Nigeria	3 (2)
Finland	3 (2)
The Netherlands	3 (2)
Japan	2 (1)
Germany	2 (1)
Korea	1 (1)
India	1 (1)
Spain	1 (1)
Iceland	1 (1)
Denmark	1 (1)
Belgium	1 (1)
Italy	1 (1)
Norway	1 (1)
Israel	1 (1)
France	1 (1)

### Level of Evidence

As shown in Table 2, four levels of evidence were adopted to show the power of evidence among included youths’ mHealth interventions for mental health research [28,29].

**Table 2:** Design of studies included in the scoping review.

Level of evidence	Study design	Articles, n
I	Randomized controlled trials	60
II	Nonrandomized experimental studies (quasi-experimental, pre-post, and cohort)	34
III	Cross-sectional, longitudinal surveys, and mixed method study with nonexperimental quantitative part	27
IV	Descriptive, case studies, and qualitative studies	30

As shown in Table 2, study types and designs varied. The highest level of evidence included experimental studies identified as randomized controlled trials (60/151, 40%). The second highest level of evidence included studies that were nonrandomized trials, such as quasi-experimental design (usually with 2 groups) or 1-group pre-post design (34/151, 23%). The third level of evidence included studies that only conducted feedback surveys to their participants once after the intervention, longitudinal observational studies, and mixed methods design with these mentioned quantitative parts (27/151, 18%). Last, some of the included studies had lower levels of evidence, including descriptive studies, case studies, and qualitative studies describing the outcome of mHealth interventions (30/151, 20%).

### Age

All included studies have a general targeted age group of “young people,” “adolescent,” or “youth.” The median age for participants in all studies was 17 (IQR 14-21) years, with 2 outliers (with participants aged 61 years [30] and 62 years [31]) coming from the studies targeting “university students.”

### Sex and Gender

A total of 20 (13%) papers did not provide any statistics on sex or gender. Only 3 (2%) studies involved participants who reported their sex or gender beyond binary options. One study focused on eating disorders and had 1 intersex participant [32]. The other 2 studies prioritized mental health in 2-spirited, lesbian, gay, bisexual, transgender, queer/questioning, intersex, and agender (2SLGBTQIA+) groups: 1 included 108 sexual minority young adults [33] and the other included 565 participants with a combination of transgender, genderqueer, gender expansive, intersex, agender, 2-spirited, and third gender identities [34]. The remaining 148 (98%) studies had a total of 6478 (34.2%) men and 12,480 (65.8%) women as participants.

### Organizational Affiliation

The included studies took place in various settings. They were coded into the following 4 different categories, inspired by Marshall et al [22]: (1) research developed under clinic or hospital-related setting (97/151, 64%); (2) research developed in a university (28/151, 19%); (3) research developed in other nonuniversity schools and institutions (9/151, 6%); and (4) insufficient information to tell (17/151, 11%). 

### Modes of Delivery of mHealth Interventions

As noted in Table 3, a variety of modes are used to deliver services. These included web pages (54/151, 36%), smartphone apps (51/151, 34%), phone calls and SMS text messages (10/151, 9%), and innovative virtual reality (VR) tools (3/151, 2%).

**Table 3:** Delivery modes among studies included in the review.

Delivery modes	Studies, n (%)	Randomized controlled trials, n/N (%)	Positive^a^ results, n/N (%)
Web pages	54 (36)	33/54 (61)	48/54 (89)
Smartphone apps	51 (34)	13/51 (26)	40/51 (78)
Video conferencing	20 (13)	3/20 (15)	15/20 (75)
SMS text messages	10 (7)	5/10 (50)	7/10 (70)
Chatbox	4 (3)	2/4 (50)	4/4 (100)
Phone calls	3 (2)	1/3 (33)	2/3 (67)
Virtual reality	3 (2)	0/3 (0)	3/3 (100)
Mixed modes	6 (4)	3/6 (50)	4/6 (67)
Total	151 (100)	60/151 (N/A^b^)	123/151 (N/A)

^a^Positive results: researchers stated overall positive effect of intervention or received positive feedback from users to show promising practical usage of the intervention. Negative results: researchers see no changes in mental health symptoms before and after the intervention or receive negative feedback from users.

^b^N/A: Not applicable.

### Targeted Conditions

The number and percentage of health conditions targeted using mHealth interventions are summarized in Table 4.

**Table 4:** Health conditions and measurement tools among included studies.

Health condition	Studies, n (%)	Measurement tools (scales) used, n
General health	26 (15)	66
Anxiety	35 (20)	52
Depression	32 (19)	68
Suicide, self-harm, or violence	11 (6)	15
Substance use	10 (6)	22
Eating disorder	10 (6)	10
Stress or mood related	9 (5)	20
Sleep disorder	6 (4)	9
ASD^a^	5 (3)	4
STD or STI^b^	5 (3)	10
Psychosis	4 (2)	5
ADHD^c^	4 (2)	8
PTSD^d^	4 (2)	3
OCD^e^	3 (2)	1
FASD^f^	2 (1)	N/A^g^
Others	6 (4)	N/A
Total	172^h^ (N/A)	N/A

^a^ASD: autism spectrum disorder.

^b^STD or STI: sexually transmitted disease or sexually transmitted infection.

^c^ADHD: attention-deficit/hyperactivity disorder.

^d^PTSD: posttraumatic stress disorder.

^e^OCD: obsessive-compulsive disorder.

^f^FASD: fetal alcohol spectrum disorder.

^g^N/A: not applicable.

^h^The total number of conditions exceeds the total number of studies because there are articles targeting more than 1 condition.

### Measurement Approaches Used in the Studies

A wide range of tools were employed to measure outcomes in mHealth intervention research (Table 4). A detailed table listing the outcome measures and the references to the studies using them can be found in Multimedia Appendix 2.

### COVID-19

Of the 151 studies included within the given time period, 68 (45%) were published after the COVID-19 outbreak, with most (44/151, 65%) published in 2020. The numbers of studies published each year are as follows: 11 studies in 2016; 21 studies in 2017; 19 studies in 2018; 32 studies in 2019; 44 studies in 2020; 23 studies in 2021; and 1 study in February 2022.

The following 8 studies directly addressed health conditions or service delivery modalities influenced by the pandemic: (1) treatment of eating disorders in adolescents [35]; (2) group-based psychiatric care using telehealth [36]; (3) a web-based art therapy group for learning disabled young adults using WhatsApp [37]; (4) telehealth versus in‐person intensive outpatient program (IOP) for eating disorders during versus before COVID‐19 [32]; (5) a well-being app to support young people living in New Zealand [38]; (6) peer-to-peer live-streaming intervention to promote physical activity and reduce anxiety during homeschooling [39]; (7) a mindfulness-based mHealth intervention among psychologically distressed university students in quarantine [40]; and (8) smartphone application for adolescents with anorexia nervosa [41].

## Discussion

### Overview

This scoping review provides a comprehensive synthesis of mobile mental health intervention research for youths. The study identified that the number of interventions is proliferating over time, with limited emphasis on study quality, youths’ engagement, youth-centered outcome assessment, implementation standards, or consideration for equity, diversity, and inclusion in research. Youths’ engagement was rarely mentioned as a part of any research study method, and most studies focused primarily on youths who identified within the gender binary. Few studies discussed the implementation and scale of interventions in diverse settings (Table 3) and even fewer studies showed an impact of mHealth interventions over time for diverse types of youths. With significant growth and investment in the area, this review may inform direction for future research and advance practices within mHealth intervention approaches for youths who experience mental health challenges or illnesses.

Among the studies that met the inclusion criteria, depression (32/151, 19%), anxiety (35/151, 20%), and general mental health concerns (26/151, 15%) accounted for over half of the studies, and the remaining addressed 17 other mental health concern categories. According to a GBD systematic analysis of mental health conditions relevant to this study, the top concerns for all youths are self-harm, depressive disorders, interpersonal violence, anxiety disorders, HIV/AIDS, conduct disorder, and drug use disorder [42]. The point estimate of alcohol and drug use disorders combined is very close to the prevalence of depression, serving as 2 of the most concerning mental health disorders worldwide [43]. The high prevalence of depressive and anxiety disorders corresponds with the allocation of research priorities in studies included in this review, suggesting the current focus for youths’ mental health care services. Nevertheless, while self-harm and interpersonal violence ranked high on the GBD mental health conditions list, there were comparatively limited research studies for these categories. Substance use disorders are also underrepresented in existing mHealth studies for youths. More attention and resources should be given to the development of mHealth intervention tools targeting self-harm, interpersonal violence, and substances use among diverse youths.

From a global health perspective, 95% (143/151) of studies came from developed countries. This result is not surprising, as mHealth resources are almost exclusively concentrated in high-income countries, although the prevalence of depression and anxiety in high-income countries is not significantly higher than in the rest of the world [44]. Therefore, more future mHealth research needs to be conducted in low- and middle-income countries, especially when one of the advantages of remote health care access is cost-effectiveness. Previous studies have demonstrated adapting interventions developed in high-income countries for use in low- and middle-income countries, such as India, Sierra Leone, Romania, Malaysia, and South Africa [45,46]; these lessons could be adapted for mHealth interventions for youths’ mental health.

### Delivery Modes

With respect to delivering mHealth interventions, most studies employed web pages (54/151, 36%) and smartphone apps (51/151, 34%), and these modes also had the largest number of randomized controlled trials conducted. The results show web-based mHealth tools have the strongest evidence for improving mental health conditions in youths, but the effectiveness of other intervention modes cannot be ruled out. Web-based intervention tools require youths to have internet access, and the use of computers may not be convenient in home and school settings, so the development of mHealth interventions has gradually evolved to include smartphones, smart devices, wearables, and newer technologies, including VR and augmented reality [47]. However, with the rising number of options available for mHealth intervention delivery, youths’ intention to use has become a more complicated question. Currently, many studies contributing to the adoption research of mHealth for youths are based on the technology acceptance model, an information systems theory that models user’s acceptance of technology mainly based on perceived usefulness and perceived ease of use [48-50]. Yet, it is essential to realize that participants in research studies are provided with a predetermined type of intervention by the researchers, often with limited youths’ engagement. That is, unlike in a real-world environment where diverse users who need support must seek it themselves, participants in the research settings did not actively choose which kind of mHealth intervention to use. Therefore, future studies need to consider the youth-intended delivery modes during mHealth intervention development from a user perspective, especially when considering innovative technologies, such as VR, artificial intelligence chat, and gaming. Future research should also consider implementation research, not only to understand the efficacy and effectiveness of the interventions but also their capacity to be sustained over time in diverse settings.

### Measurement

The measurement tools used in the studies (Multimedia Appendix 2) can be broadly defined as either (1) measuring the outcome of a patient’s condition or (2) measuring the subject usability of a product or service. As displayed in Table 4, depression, anxiety, and general mental health categories each adopted more than 50 measurement tools. The review also identified numerous measurement scales used for each health condition. For example, 7 sleep-relevant measurements were used in 6 sleep-related studies [51-56], but the researchers did not provide a rationale as to why they chose the scale used among all available sleep-related measurements, nor was evidence provided about the fitness of the tools for youths in the varying contexts. Similar complications were presented in other included studies with varying health conditions. Future research should focus on developing a guideline for researchers to follow when selecting the most appropriate measurement scales in both research and clinical settings and on validating measurement scales designed for use with youths with consideration for equity, diversity, inclusion, and psychometric rigor.

### Evaluation of mHealth Products

The percentage of positive results is considerably high among all included studies (123/151, 81%). Study aims included “feasibility,” “effect,” “acceptability,” “efficacy,” “fidelity,” “effectiveness,” and “cost-effectiveness,” with research designs including experiments, surveys, and interviews. Questions remain as to whether positive results translate to real-world applications or which types of outcomes possess high level of evidence in the knowledge translation process for health care services. In a Canadian study where participants were asked to rate mHealth apps objectively, results were highly variable, and 28% of reviewers were not even sure about the overall quality of the health product [57]. The same characteristics in different studies also vary within a wide range. For instance, the intervention durations ranged from as short as 7 days [58] or 2 sessions [59] to as long as 24 weeks [60] or 24 months [61]. The number of participants among all studies ranges from 2 [62] to 2532 [63]. Intervention types also varied significantly. Such variations raise concerns about the lack of a standardized evaluation strategy. To address the complex uncertainty in evaluating mHealth tools, a multifaceted evaluation framework needs to be adopted to assess the different perspectives, elements, and features of an intervention that leads to a final mHealth product. Using non–youth-centered frameworks to evaluate mHealth products can consequently produce ambiguous or incorrect information on their effectiveness, leading to misuse, misdiagnosis, wasted time, and, worst of all, negative health impacts and experiences [64].

### Remote Care Transition

Several included studies discuss how to support youths during the COVID-19 pandemic [65,66]. With many people transitioning to work-from-home or hybrid arrangements during and after the pandemic, there arose an imminent need for a mental health technology revolution, and web-based health service delivery has emerged as a preferred tool [67].

Childs and colleagues [36] illustrated the feasibility of a rapid transition to telemedicine services during the pandemic. Yale New Haven Psychiatric Hospital decided to discontinue in-person IOP services within 3 business days of the World Health Organization’s pandemic declaration [36]. The first mobile service was available within a week, and subsequent treatment plans and adolescent ambulatory services were developed to reach IOP level. The study demonstrated that it took a comprehensive program 2 months to transition from 100% in-person service prepandemic (March) to 100% telehealth service after the start of the pandemic (May 2020), showing the feasibility of the deployment of mHealth tools in clinical settings and the smooth transition from physical to virtual health care access. One notable limitation of this study is that the clinicians focused on the transition process rather than the effectiveness of the intervention tool.

Another youths’ mHealth intervention study showed the transition to virtual services was not always desired by clients. The authors presented a case where a participant refused to cooperate, and the telemedicine service increased the tension between the participant and family members [35]. When developing mHealth interventions targeting youths’ mental health, it is important to consider how to achieve optimal patient engagement when physical contact with a service provider is not an option. Last, it is still unknown whether these transitioned services will continue to be provided virtually on an ongoing basis. Future research is needed to investigate the long-term influence of such transitions and determine the feasibility of normalizing mHealth services.

### Youths’ and Stakeholder Engagement

Engagement with mHealth interventions is thought to be important for intervention effectiveness by increasing acceptability, satisfaction, intervention adherence, and levels of attention and enjoyment [68]. This can be extended to engaging youths in mHealth research to provide comprehensive, ongoing, tailored, and interactive support to improve health [69].

Current youth mHealth research often engages youths by asking for feedback in a survey or interview to test usability, feasibility, and acceptability [31,70,71], or by including young users as participants in experiments to evaluate the effectiveness and efficiency of an intervention. However, few studies mentioned how they engaged youths in the development phase and followed design thinking with the priority population. Youths and other stakeholders (eg, family and caregivers, service providers, and graphic designers) can contribute more than just feedback on the provided services; they can be offered opportunities to participate in the product and service design stages to make sure the end product is tailored to their needs and preferences. A previous conceptual model indicated that, during the optimization phase of an intervention [72], participants need to understand how the provided materials can inspire them and facilitate their thoughts to improve self-efficacy, which increases capability for self-monitoring and self-regulation and can lead to improved health outcomes and behavioral changes [72,73]. Researchers proposed a supportive accountability model that emphasized the importance of human support in mHealth interventions to increase adherence to trustworthy, benevolent, and professional information [74]. To summarize, it is crucial to apply such theoretical models to interventions targeting youths’ mental health as well. mHealth researchers and developers ought to involve youths in every stage of design, development, and implementation. Our team is currently studying youths’ engagement in the mHealth development phase to understand youths’ information preferences and make sure mHealth interventions are designed to convey the benefits of human support, similar to in-person services.

### Policy

More than two-thirds of the studies took place in a clinical setting, yet none of the studies reviewed provided systematic frameworks or models to help translate, scale, and sustain available mHealth tools to clinical practice. If health care stakeholders and policy makers aim to scale up and normalize mHealth services in the near future, it is essential to understand the feasibility and impact of implementing new mHealth tools in current models of care (eg, health care and schools). Guidelines and standards may be critical to ensuring that mHealth interventions are trustworthy and can be value added to health services that are delivered to youths and their families.

### Strengths and Limitations

This review has a broad scope of attempting to draw a picture of existing mHealth intervention tools specifically designed for younger populations and how their effectiveness is being assessed. This scoping review addressed a broad term list and a large number of parameters. Inclusion and exclusion criteria were strictly set from the beginning and determined by experts in the field and a medical librarian, and diversity is presented for all included studies. There are unlimited possibilities for future work, particularly with the uncertainty of the COVID-19 pandemic and the ongoing response of the health care system to remote health access. Regarding weaknesses, the lack of critical appraisal is a widely recognized limitation for scoping reviews [75]. The scope of this review may be broad, but the depth and the quality of all included papers were not systematically critiqued. We also acknowledge that our search strategy may have missed key terms (eg, internet-based interventions) and intervention descriptions (eg, asynchronous vs synchronous) that may have limited our ability to completely summarize all relevant articles. In addition, the COVID-19 pandemic has not come to an end, and there is still ongoing research about long-term COVID-19 symptoms. Thus, the results relevant to remote care transition and COVID-19 should be interpreted with some caution.

### Conclusions

As the need for mental health services continues to accelerate [76], mHealth technologies provide a solution to support diverse youths who may not be able to access in-person services. The impact of mHealth interventions on youths’ mental health has been increasingly recognized by researchers, service providers, and policy makers. Results of our scoping review demonstrate a range of studies that capture the exponential growth of mHealth interventions for youths, with significant potential to be value-added for youths who are seeking support for mental health challenges. However, the review also highlighted notable gaps in research that include youths’ voice throughout the research process, notably diverse youths in both developing and developed countries. Future research is needed that adopts an equity, diversity, and inclusion lens, prioritizes understanding how current mHealth technologies can be adopted into existing models of care, and develops guidelines, standards, and evaluation frameworks to support future mHealth development and implementation. As the field continues to expand rapidly, more global resources are needed to monitor technological advancements to provide quality mHealth services to every youth where and when they need them.

## Appendix

Multimedia Appendix 1 Sample Literature Search Sheet.

Multimedia Appendix 2 List of measurement scales and studies using them.

## Abbreviations

2SLGBTQIA+: 2-spirited, lesbian, gay, bisexual, transgender, queer/questioning, intersex, and agender
GBD: global burden of disease
IOP: intensive outpatient program
MeSH: Medical Subject Heading
mHealth: mobile health
VR: virtual reality
