# Supplementary material for: Understanding Mobile Health and Youth Mental Health: Scoping Review
Source: JMIR Mhealth Uhealth. 2023 Jun 16;11:e44951. doi: 10.2196/44951 (PMC10278734; doi:10.2196/44951)
Supplement: Multimedia Appendix 1 [file mhealth_v11i1e44951_app1.docx]

**Appendix A Sample Literature Search Sheet**

Embase <2016 to 2022 February 07>

1 telemedicine/ or telerehabilitation/ 37099

2 mobile application/ 16795

3 (mhealth or telemedicine or telehealth or mobile health or ehealth or electronic health).mp. [mp=title, abstract, heading word, drug trade name, original title, device manufacturer, drug manufacturer, device trade name, keyword heading word, floating subheading word, candidate term word] 105313

4 1 or 2 or 3 118417

5 adolescent/ 1646536

6 (teen* or adolescent* or youth* or young adult).mp. [mp=title, abstract, heading word, drug trade name, original title, device manufacturer, drug manufacturer, device trade name, keyword heading word, floating subheading word, candidate term word] 2079194

7 5 or 6 2079194

8 mental disease/ 241422

9 (anxiety or depression or eating disorder* or schizophrenia or bipolar or obsessive compulsive disorder or posttraumatic stress disorder or post traumatic stress disorder).mp. [mp=title, abstract, heading word, drug trade name, original title, device manufacturer, drug manufacturer, device trade name, keyword heading word, floating subheading word, candidate term word] 1264854

10 8 or 9 1409947

11 4 and 7 and 10 1344
