# Supplementary material for: Understanding Mobile Health and Youth Mental Health: Scoping Review
Source: JMIR Mhealth Uhealth. 2023 Jun 16;11:e44951. doi: 10.2196/44951 (PMC10278734; doi:10.2196/44951)
Supplement: Multimedia Appendix 2 [file mhealth_v11i1e44951_app2.docx]

**Appendix B List of measurement scales and studies using them**

*Depression, anxiety, and general mental health scales*

|  | Depression | Anxiety | General Mental Health |
| --- | --- | --- | --- |
| 10 Usability Heuristics for User Interface Design - Nielsen |  | Newton et al., 2020 |  |
| A Stop Smoking in Schools Trial (ASSIST) |  |  | Thabrew et al., 2020 |
| Acculturation Rating Scale for Mexican Americans-II (ARSMA-II) |  | Chavira et al., 2018 |  |
| Achenbach System of Empirically Based Assessment (ASEBA) |  |  | Campbell et al., 2019 |
| Anxiety Disorders Interview Schedule for Children and Parents (ADIS-C/P) |  | Carpenter et al., 2018; Donovan et al., 2017; Stasiak et al., 2018; Stjerneklar et al., 2019; Spence et al., 2020; Chavira et al., 2018; McLellan et al., 2017; Jolstedt et al., 2020 |  |
| Adolescent Life Events Questionnaire (ALEQ) | Van Voorhees et al., 2020 |  |  |
| Adverse Childhood Experience (ACE) Questionnaire | MacIsaac et al., 2021 |  |  |
| Alcohol Use Disorders Identification (AUDIT) | Hides et al., 2019 |  | Pachankis et al., 2020; Ospina-Pinillos et al., 2018 |
| Alcohol, Smoking and Substance Involvement Screening Test |  |  | Ospina-Pinillos et al., 2018 |
| Altman Self-Rating Mania Scale (ASRM) |  |  | Ospina-Pinillos et al., 2018 |
| Anxiety Literacy Questionnaire |  |  | Lattie et al., 2020 |
| Avoidance and Fusion Questionnaire for Youth (AFQ-Y) |  |  | Lappalainen et al., 2021 |
| Barriers to Treatment Participation Scale |  | Carpenter et al., 2018; Chavira et al., 2018 |  |
| Basic Psychological Need Satisfaction Scales |  |  | Alvarez-Jimenez et al., 2020 |
| Beck Anxiety Inventory | Topooco et al., 2019), (Ahmad et al., 2020), (Topooco et al., 2018) | (Short & Schmidt, 2020) | (Pachankis et al., 2020) |
| Beck Depression Inventory II | (Ofoegbu et al., 2020), (Ritvo et al., 2021), (Ranney et al., 2018) (Topooco et al., 2019), (Topooco et al., 2018) |  |  |
| Beck Hopelessness Scale | (Van Voorhees et al., 2020) |  |  |
| Brief Pain Inventory (BPI) | (Ritvo et al., 2021) |  |  |
| Brief Symptom Inventory (BSI) |  |  | (Pachankis et al., 2020) |
| Brunnsviken Brief Quality of life scale (BBQ) | (Topooco et al., 2019) |  |  |
| Center for Epidemiologic Studies Depression Scale (CESD-10) | (Van Voorhees et al., 2020), (Bansa et al., 2018), 1(Ranney et al., 2018) | (Donovan et al., 2017) | 7, (Campbell et al., 2019), (Pachankis et al., 2020) |
| Client Evaluation Questionnaire (CEQ) |  | (Short & Schmidt, 2020) |  |
| Child Anxiety Life Inference Scale (CALIS) |  | (Stjerneklar et al., 2019), (McLellan et al., 2017) |  |
| Child Behavior Checklist (CBCL) |  | (Conaughton et al., 2017), (Donovan et al., 2017) |  |
| Child Health Utility 9D (CHU9D) |  | (Stasiak et al., 2018) |  |
| Child Report of Parental Behavior Inventory | (Van Voorhees et al., 2020) |  |  |
| Children's Anxiety Scale |  | (March et al., 2018), |  |
| Children's Depression Rating Scale-Revised (CDRS-R) | (Whittaker et al., 2017) |  |  |
| Children’s Depression Inventory (CDI) |  | (Kwon et al., 2020) | (Egilsson et al., 2021) |
| Children’s Global Assessment Scale (C-GAS) | (Bansa et al., 2018) | (Conaughton et al., 2017), (Donovan et al., 2017), (Stasiak et al., 2018), (Jolstedt et al., 2020), |  |
| Client Satisfaction Questionnaire (CSQ-8) | (Ranney et al., 2018) | (Carpenter et al., 2018), (March et al., 2018), (Newton et al., 2020), | (Le et al., 2019) |
| Clinical Global Impression – Improvement scale (CGI-I) | (Rice et al., 2018) |  |  |
| Clinical Severity Rating (CSR) |  | (Carpenter et al., 2018), (Stjerneklar et al., 2019), (Jolstedt et al., 2020) |  |
| Cognitive and Behavioral Response to Stress Scale (CB-RSS) |  |  | (Lattie et al., 2020) |
| Cognitive Therapy Rating Scale (CTRS) | (Ranney et al., 2018) |  |  |
| Colorado Symptom Index (CSI) |  |  | (Brunette et al., 2018) |
| Connor-Davidson Resilience Scale 10-Item | (MacIsaac et al., 2021) |  |  |
| Credibility/Expectancy Questionnaire (CEQ) | (Topooco et al., 2019) |  |  |
| Daily Drinking Questionnaire (DDQ) |  |  | (Viskovich & Pakenham, 2018) |
| Daily Drug Taking Questionnaire |  |  | (Viskovich & Pakenham, 2018) |
| Depression, Anxiety, and Stress Scale (DASS-21) | (Nguyen-Feng et al., 2017), (Rice et al., 2018) | (Hall et al., 2018), | (Viskovich & Pakenham, 2018), |
| Depression Literacy Questionnaire (D-Lit) |  |  | (Lattie et al., 2020), |
| Depression Scale (DEPS) |  |  | (Lappalainen et al., 2021) |
| Difficulties in Emotion Regulation Scale (DERS) | (MacIsaac et al., 2021), (Lindqvist et al., 2020) |  | (Schueller et al., 2019) |
| Dimensional Obsessive-Compulsive Scale |  | (Short & Schmidt, 2020) |  |
| Distress Questionnaire 5 (DQ-5) | (Anttila et al., 2020) |  |  |
| Drinking Motives Questionnaire-Revised (DMQR) |  |  | (Ospina-Pinillos et al., 2018) |
| Early Adolescent Temperament Questionnaire (EATQ-R) |  |  | (Stormshak et al., 2019), |
| Engaged Living Scale (ELS) |  |  | (Viskovich & Pakenham, 2018) |
| EPOCH (engagement, perseverance, optimism, connectedness, and happiness) Measure of Adolescent Well-being | (Osborn et al., 2020) |  |  |
| EUROHIS-QOL 8-item index (a shortened version of the World Health Organization  Quality of Life Instrument-Abbreviated Version) | (Raevuori et al., 2021) |  |  |
| Executive Function Index (EFI) | (MacIsaac et al., 2021) |  |  |
| Experience of Service Questionnaire (ESQ) |  | (Stjerneklar et al., 2019) |  |
| Fagerström Test for Nicotine Dependence |  |  | (Brunette et al., 2018), (Ospina-Pinillos et al., 2018) |
| Fear of Negative Evaluation Scale (FNE) |  | (McCall et al., 2018) |  |
| Five Well-Being Index (WHO-5) |  | (Stjerneklar et al., 2019) | (Arps et al., 2018) |
| Flexibility Index Test (FIT-60) | (van Aubel et al., 2020) |  | (Lattie et al., 2020), |
| Freiburg Mindfulness Inventory |  |  | (Alvarez-Jimenez et al., 2020) |
| Friendship Scale |  |  | (Alvarez-Jimenez et al., 2020) |
| Generalized Anxiety Disorder Assessment (GAD-7) | (Raevuori et al., 2021), (Dear et al., 2019), (Staples et al., 2019), (Lindqvist et al., 2020), (Cook et al., 2019), (Osborn et al., 2020), (Dear et al., 2018) |  | (Arps et al., 2018), (Thabrew et al., 2019), (Thabrew et al., 2020) |
| General Health Questionnaire |  |  | (Kajitani et al., 2020), (Sekizaki et al., 2019) |
| General Help Seeking Questionnaire (GHSQ) | (Anttila et al., 2020), (Wiljer et al., 2020) |  | (Le et al., 2019) |
| General Self-Efficacy Scale (GSE) | (Topooco et al., 2019), (Topooco et al., 2018) |  | (Egilsson et al., 2021), (Sekizaki et al., 2019) |
| Global Assessment Scale (GAS) | (Van Voorhees et al., 2020) |  |  |
| Hamilton Depression Rating Scale (HDRS) | (Ritvo et al., 2021) |  |  |
| Hardship Index | (Bansa et al., 2018) |  |  |
| HIV Risk-taking Behaviour Scale (HRBS) |  |  | (Pachankis et al., 2020) |
| Hospital Anxiety and Depression Scale (HADS) | (McCloud et al., 2020) |  | (Bendtsen et al., 2020) |
| Insomnia Severity Index | (Raevuori et al., 2021) |  |  |
| International Physical Activity Questionnaire |  |  | (Ospina-Pinillos et al., 2018) |
| Kessler Psychological Distress Scale (K10) | (Staples et al., 2019), (Dear et al., 2018) |  | 17, (Le et al., 2019), (Ospina-Pinillos et al., 2018) |
| K-SADS-PL (Present and Lifetime Version) |  | (Silk et al., 2020) |  |
| Kessler Psychological Distress Scale (K6) |  |  | (Sekizaki et al., 2019) |
| Kiddie-Schedule for Affective Disorders and Schizophrenia for School-Age Children (K-SADS) | (Van Voorhees et al., 2020), (Whittaker et al., 2017), (Bansa et al., 2018) |  |  |
| KIDSCREEN-10 (C/P) |  | (Jolstedt et al., 2020) |  |
| Knowledge and Beliefs about Services Scale (KBSS) |  |  | (Lattie et al., 2020) |
| Korean Test Anxiety Inventory |  | (Kwon et al., 2020) |  |
| Mental Health Continuum Short Form (MHC-SF) |  |  | (Viskovich & Pakenham, 2018), (Bendtsen et al., 2020) |
| Mental Help Seeking Intention Scale (MHSIS) | (Cynthia Logsdon et al., 2018) |  |  |
| Mindful Attention Awareness Scale |  |  | (Viskovich & Pakenham, 2018) |
| Montgomery-Asberg Depression Rating Scale (MADRS) | (Rice et al., 2018), (van Aubel et al., 2020), (Lindqvist et al., 2020) |  |  |
| Moods and Feelings Questionnaire (MFQ) | (Whittaker et al., 2017), (Topooco et al., 2019) | (Stasiak et al., 2018), (Stjerneklar et al., 2019), (Yap et al., 2019), (McLellan et al., 2017) |  |
| Multidimensional Anxiety Scale for Children (MASC) |  |  | (Egilsson et al., 2021) |
| Multidimensional Scale of Perceived Social Support |  |  | (Campbell et al., 2019) |
| Multidimensional Student's Life Satisfaction Scale (MSLSS) | (Ahmad et al., 2020) |  |  |
| National Survey on Drug Use and Health (NSDUH) |  |  | (Ospina-Pinillos et al., 2018) |
| Outcome Rating Scale (ORS) |  | (Chapman et al., 2016) |  |
| Parent Consumer Satisfaction Scale |  | (Chavira et al., 2018) |  |
| Parenting Tasks Checklist |  |  | (Stormshak et al., 2019) |
| Parenting to Reduce Adolescent Depression and Anxiety Scale (PRADAS) |  | (Yap et al., 2019) |  |
| Pediatric Quality of Life Enjoyment and Satisfaction Questionnaire (PQ-LES-Q) | (Whittaker et al., 2017) |  |  |
| Penn State Worry Questionnaire | (Rice et al., 2018), (Cook et al., 2019) | (Short & Schmidt, 2020) |  |
| Perceived Stress Scale (PSS) | (Nguyen-Feng et al., 2017), (Raevuori et al., 2021), (Ahmad et al., 2020) |  | (Alvarez-Jimenez et al., 2020) |
| Personal Values Questionnaire (PVQ) |  |  | (Viskovich & Pakenham, 2018) |
| PHQ-2 (2 items from PHQ-9) | (Bansa et al., 2018) (PHQ2A), (Cynthia Logsdon et al., 2018) |  |  |
| PHQ-8 (8 items from PHQ-9) | (Osborn et al., 2020) |  | (Lattie et al., 2020), |
| Patient Health Questionnaire (PHQ-9) | (MacIsaac et al., 2021), (Ranney et al., 2018), (Hides et al., 2019), (Dear et al., 2019), (Staples et al., 2019), (Ahmad et al., 2020), (Cook et al., 2019), (Dear et al., 2018), (Topooco et al., 2018) | (Chen et al., 2017) | (Schueller et al., 2019), (Alvarez-Jimenez et al., 2020), (Arps et al., 2018) |
| PHQ-A (PHQ-9 modified for Adolescents) | (Anttila et al., 2020), (Van Voorhees et al., 2020) |  | (Thabrew et al., 2019), (Thabrew et al., 2020) |
| PTSD Checklist for DSM-5 (PCL-5) |  | (Short & Schmidt, 2020), | (Schueller et al., 2019) |
| Quality of Life Enjoyment and Satisfaction Questionnaire - Short Form (Q-LES-Q-SF) |  | (McCall et al., 2018) |  |
| Quality of Life Scale (QOLS) | (Ahmad et al., 2020) |  | (Le et al., 2019) |
| Quick Inventory of Depressive Symptomatology (QIDS) | (Ritvo et al., 2021), (Lindqvist et al., 2020) |  |  |
| Reactions to Program Scale (RPS) |  | (Stoll et al., 2017) |  |
| Resilience Scale | (Raevuori et al., 2021) |  |  |
| Revised Children's Anxiety and Depression Scale (RCADS) |  | (Jolstedt et al., 2020), (Chapman et al., 2016) |  |
| Reynold’s Adolescent Depression Rating Scale – 2nd Edition (RADS-2) | (Whittaker et al., 2017) |  |  |
| Rosenberg Self-Esteem Scale |  |  | (Arps et al., 2018), (Campbell et al., 2019) |
| Ruminative Response Scale (RRS) | (Cook et al., 2019) |  |  |
| Safety Aid Scale (SAS) |  | (Short & Schmidt, 2020) |  |
| Satisfaction with Life Scale |  |  | (Arps et al., 2018), (Viskovich & Pakenham, 2018), (Lappalainen et al., 2021) |
| Screen for Child Anxiety Related Disorders (SCARED) |  | (Hepburn et al., 2016) |  |
| SELF-COMPASSION SCALE–Short Form (SCS–SF) | (Lindqvist et al., 2020) |  | (Viskovich & Pakenham, 2018) |
| Self-Efficacy Questionnaire for Children (SEQ-C) | (Anttila et al., 2020) | (Stjerneklar et al., 2019) |  |
| Sheehan Disability Scale (SDS) | (Dear et al., 2018) |  |  |
| Social Interaction Anxiety Scale (SIAS) | (Topooco et al., 2019) | (McCall et al., 2018), |  |
| Social Occupational Functioning Scale (SOFS) | (Rice et al., 2018) |  |  |
| Social Responsiveness Scale |  | (Hepburn et al., 2016) |  |
| Social Support Survey | (Rice et al., 2018) |  |  |
| Somatic and Psychological HEalth REport (SPHERE) |  |  | (Ospina-Pinillos et al., 2018) |
| Spence Children's Anxiety Scale (SCAS) | (Anttila et al., 2020) | (Conaughton et al., 2017), (Stasiak et al., 2018), (Stjerneklar et al., 2019), (Yap et al., 2019), (Spence et al., 2020), (McLellan et al., 2017), |  |
| Stanford-Binet Intelligence Scales (SB-5) |  | (Hepburn et al., 2016) |  |
| State-Trait Anxiety Inventory (STAI) |  | (Kenny et al., 2020) |  |
| Stigma Scale |  |  | (Kajitani et al., 2020) |
| Stigma Scale for Receiving Psychological Help (SSRPH) | (Cynthia Logsdon et al., 2018) |  |  |
| Strengths and Difficulties Questionnaire (SDQ) |  | (McLellan et al., 2017) | (Stormshak et al., 2019) |
| Strengths Use Scale (SUS) | (Rice et al., 2018) |  | (Alvarez-Jimenez et al., 2020) |
| Substances and Choices Scale (SACS) |  |  | (Thabrew et al., 2020) |
| Suicidal Ideation Attributes Scale (SIDAS) |  |  | (Pachankis et al., 2020), (Ospina-Pinillos et al., 2018) |
| Symptom Checklist (SCL-90) | (van Aubel et al., 2020), |  | (Van Dam et al., 2019), |
| System Usability Scale (SUS) | (Raevuori et al., 2021) | (Kanuri et al., 2020), (Newton et al., 2020) | (Egilsson et al., 2021) |
| Five Facet Mindfulness Questionnaire (FFMQ) | (Ritvo et al., 2021), (Raevuori et al., 2021), (Ahmad et al., 2020) |  |  |
| Gratitude Questionnaire – Six Item Form (GQ-6) |  |  | (Arps et al., 2018) |
| The Inventory for Depressive Symptomatology (IDS) | (van Aubel et al., 2020) |  |  |
| Motivational Interviewing Treatment Integrity (MITI) | (van Voorhees et al., 2020) |  |  |
| The Short Inventory of Problems-Modified for Drug Use (SIP-DU) |  |  | (Pachankis et al., 2020) |
| State-Trait Anxiety Inventory (STAI) | (van Aubel et al., 2020) |  |  |
| Theoretical Domains Framework (TDF) |  | (Newton et al., 2020) |  |
| Traumatic Life Events Questionnaire (TLEQ) | (Nguyen-Feng et al., 2017) |  |  |
| Treatment Adherence Questionnaire |  | (Chavira et al., 2018) |  |
| Treatment Satisfaction and Acceptability Measure (TSAM) |  | (Fitzsimmons-Craft et al., 2019) |  |
| UCLA Loneliness Scale |  |  | (Alvarez-Jimenez et al., 2020) |
| Usefulness, Satisfaction, and Ease of use (USE Questionnaire) |  | (Stoll et al., 2017) |  |
| Visual Analogue Scale (VAS) |  | (Kwon et al., 2020) |  |
| Warwick–Edinburgh Mental Wellbeing Scale | (Anttila et al., 2020), (Osborn et al., 2020) |  | (Alvarez-Jimenez et al., 2020) |
| Wechsler Intelligence Scale for Children (WISC-V) |  | (Kwon et al., 2020), (Hepburn et al., 2016), |  |
| Work and Social Adjustment Scale (WSAS) Child/Parent version (C/P) |  | (Jolstedt et al., 2020) |  |
| Working Alliance Inventory – short version (WAI-S) | (Topooco et al., 2019) | (Carpenter et al., 2018) |  |
| Youth Risk Behaviour Survey | (Whittaker et al., 2017) |  |  |

*Additional conditions*

| Violence/self-harm/suicide | Conflict Tactics Scales (CTS) | (Ranney et al., 2018) |
| --- | --- | --- |
|  | Columbia Suicide Severity Rating Scale (C-SSRS) | (Rengasamy & Sparks, 2019) |
|  | Depressive Symptom Index-Suicidality Subscale (DSI-SS) | (Tighe et al., 2020), (Tighe et al., 2017) |
|  | PHQ-9 | (Tighe et al., 2020), (Tighe et al., 2017) |
|  | K10 | (Tighe et al., 2020), (Tighe et al., 2017), (Iorfino et al., 2017) |
|  | International Classification of Diseases | (Fairchild et al., 2020) |
|  | Beck Depression Inventory‐II | (Wright et al., 2021) |
|  | Barratt Impulsiveness Scale (BIS-11) | (Tighe et al., 2017) |
|  | Suicidal Ideation Attributes Scale (SIDAS) | (Iorfino et al., 2017) |
|  | Brief Disability Questionnaire (BDQ) | (Iorfino et al., 2017) |
|  | Alcohol Use Disorders Identification Test (AUDIT) | (Iorfino et al., 2017) |
|  | Strengths and Difficulties Questionnaire | (Stallard et al., 2018) |
|  | Mood and Feelings Questionnaire (MFQ) | (Stallard et al., 2018) |
|  | The Revised Child Anxiety and Depression Scale (RCADS) | (Stallard et al., 2018) |
|  | Symptom Checklist (SCL-90) | (van Rosmalen-Nooijens et al., 2017) |
|  | | |
| Substance | PHQ-2 | (Mason, 2020) |
|  | AUDIT | (Saberi et al., 2021), (King et al., 2020), (Coughlin et al., 2021) |
|  | DAST | (Saberi et al., 2021) |
|  | ASSIST | (Saberi et al., 2021) |
|  | GAD-7 | (Saberi et al., 2021) |
|  | PHQ-9 | (Saberi et al., 2021) |
|  | PCL-50 | (Saberi et al., 2021) |
|  | HIV Treatment Knowledge Scale | (Saberi et al., 2021) |
|  | Rutgers Alcohol Problems Index (RAPI) | (King et al., 2020) |
|  | Desire for Drug Questionnaire (DDQ) | (King et al., 2020) |
|  | Motivational Interviewing Treatment Integrity | (King et al., 2020) |
|  | Client Satisfaction Questionnaire (CSQ-8) | (King et al., 2020) |
|  | Temple Presence Inventory | (King et al., 2020) |
|  | Beck Depression Inventory | (McClure et al., 2018) |
|  | Alcohol Timeline FollowBack (TLFB) | (McClure et al., 2018) |
|  | Fagerström Tolerance Questionnaire (FTQ) | (McClure et al., 2018) |
|  | Daily Drinking Questionnaire | (Kazemi et al., 2019) |
|  | Substance Use Risk Profile Scale (SURPS) | (Hides et al., 2021) |
|  | Usefulness, Satisfaction, and Ease of Use Questionnaire (USE) | (Kazemi et al., 2019) |
|  | The Short Inventory of Problems – Revised (SIP-R) | (Braciszewski et al., 2018) |
|  | Brief Situational Confidence Questionnaire (BSCQ) | (Braciszewski et al., 2018) |
|  | Center for Epidemiologic Studies Depression Scale (CES-D) | (Braciszewski et al., 2018) |
|  | | |
| Psychosis | Community Assessment of Psychic Experiences (CAPE) | (van Aubel et al., 2020) |
|  | Brief Psychiatric Rating Scales (BPRS) | (Niendam et al., 2018) |
|  | Children’s Global Assessment Scale (CGAS) | (Smelror et al., 2019) |
|  | Wechsler Abbreviated Scale of Intelligence | (Smelror et al., 2019) |
|  | Beliefs About Voices Questionnaire-Revised version (BAVQ-R) | (Smelror et al., 2019) |
|  | | |
| Sleep | Pittsburgh Sleep Quality Index (PSQI) | (Hall et al., 2018), (Werner-Seidler et al., 2019), (Gipson et al., 2019) |
|  | Insomnia Severity Index | (Werner-Seidler et al., 2019), (Short & Schmidt, 2020) |
|  | Sleep-Related Behaviors Questionnaire (SRBQ) | (Short & Schmidt, 2020) |
|  | Holland Sleep Disorders Questionnaire | (de Bruin et al., 2020) |
|  | The Chronic Sleep Reduction Questionnaire (CSRQ) | (de Bruin et al., 2020) |
|  | Sleep Hygiene Awareness and Practice Scale | (Gipson et al., 2019) |
|  | Sleep Hygiene Index (SHI) | (Gipson et al., 2019) |
|  | PHQ-A | (Werner-Seidler et al., 2019) |
|  | GAD-7 | (Werner-Seidler et al., 2019) |
|  | | |
| ASD | Childhood Asperger Syndrome Test | (Conaughton et al., 2017) |
|  | Autism Diagnostic Interview-Revised (ADI-R) | (Hepburn et al., 2016) |
|  | Social Responsiveness Scale-2nd Edition (SRS-2) | (Smith et al., 2020) |
|  | Treatment Credibility Scale (TCS) | (Backman et al., 2018) |
|  | | |
| OCD | Children Yale-Brown Obsessive Compulsive Scale (CY-BOCS) | (Tie et al., 2019), (Lenhard et al., 2017) |
|  | | |
| ADHD | Vanderbilt ADHD Diagnostic Rating Scale (VADRS) | (Schoenfelder et al., 2017), (Rockhill et al., 2016) |
|  | Positive and Negative Affect Schedule (PANAS) | (Schoenfelder et al., 2017) |
|  | CSQ-8 | (Schoenfelder et al., 2017) |
|  | Impairment Rating Scale (IRS) | (Kollins et al., 2021) |
|  | ADHD Rating Scale-IV (ADHD-RS) | (Kollins et al., 2021) |
|  | Clinical Global Impression (CGI) | (Kollins et al., 2021) |
|  | Computerized Diagnostic Interview Schedule for Children (C-DISC) | (Rockhill et al., 2016) |
|  | Child Behavior Checklist (CBCL) | (Rockhill et al., 2016) |
|  |  |  |
| Stress/Mood/Emotion/Loneliness | PHQ-9 | (Saberi et al., 2020) |
|  | PTSD Checklist for DSM-5 (PCL-5) | (Saberi et al., 2020) |
|  | Adverse Childhood Experiences Questionnaire (ACE) | (Saberi et al., 2020) |
|  | ASSIST Alcohol, Smoking and Substance Involvement Screening Test | (Saberi et al., 2020) |
|  | DAST Drug Abuse Screening Test | (Saberi et al., 2020) |
|  | AUDIT | (Saberi et al., 2020) |
|  | DDS Diabetes Distress Scale | (Bakhach et al., 2019) |
|  | Self-Efficacy for Diabetes Scale | (Bakhach et al., 2019) |
|  | Self-Management of Type 1 Diabetes in Adolescence (the SMOD-A) | (Bakhach et al., 2019) |
|  | Center for Epidemiologic Studies Depression Scale (CES-D) | (Bakhach et al., 2019), (Lim et al., 2019) |
|  | Difficulties in Emotion Regulation Scale-SF (DERS-SF) | (Hides et al., 2019) |
|  | Mental Health Continuum Short Form (MHC-SF) | (Hides et al., 2019) |
|  | K10 | (Hides et al., 2019) |
|  | Healthy-Unhealthy Music Scale (HUMS) | (Hides et al., 2019) |
|  | UCLA Loneliness Scale (UCLA-LS) | (Lim et al., 2019) |
|  | Social Interaction Anxiety Scale (SIAS) | (Lim et al., 2019) |
|  | Young Mania Rating Scale | (Miklowitz et al., 2021) |
|  | CDRS-R | (Miklowitz et al., 2021) |
|  | Parent General Behavior Inventory (P-GBI) | (Miklowitz et al., 2021) |
|  | Children's Affective Lability Scale (CALS) | (Miklowitz et al., 2021) |
|  | | |
| PTSD | UCLA Posttraumatic Stress Disorder Reaction Index (UCLA PTSD-RI) | (Stewart et al., 2021), (Stewart et al., 2020) |
|  | Short Mood and Feelings Questionnaire (SMFQ) | (Stewart et al., 2021), (Stewart et al., 2020) |
|  | Telehealth Satisfaction Questionnaire (TSQ) | (Stewart et al., 2021) |
|  | | |
| STD | The MOS Social Support Survey | (Dulli et al., 2020) |
|  | AIDS Clinical Trials Group Adherence Questionnaire | (Dulli et al., 2020) |
|  | 4-item PROMISc Social Isolation Scale | (Dulli et al., 2020) |
|  | PHQ-8 | (Dulli et al., 2020) |
|  | HIV Stigma Scale | (Dulli et al., 2020) |
|  | DAST | (Lopez et al., 2020) |
|  | AUDIT | (Lopez et al., 2020) |
|  | UCLA-RI | (Lopez et al., 2020) |
|  | Emotion Regulation Questionnaire (ERQ) | (Lopez et al., 2020) |
|  | Difficulties in Emotion Regulation Scale (DERS) | (Lopez et al., 2020) |
|  |  |  |
| ED | Weight Concerns Scale (WCS) | (Fitzsimmons-Craft et al., 2019) |
|  | Eating Disorder Examination Questionnaire (EDE-Q) | (Fitzsimmons-Craft et al., 2019), (Shu et al., 2019), (van Aubel et al., 2020), (Levinson et al., 2021) |
|  | Eating Disorder Diagnostic Scale (EDDS) | (Fitzsimmons-Craft et al., 2019) |
|  | System Usability Scale (SUS) | (Anastasiadou et al., 2019) |
|  | CSQ-8 | (Anastasiadou et al., 2019) |
|  | Clinical Perfectionism Questionnaire (CPQ) | (Shu et al., 2019) |
|  | Revised Children's Anxiety and Depression Scale (RCADS) | (Shu et al., 2019) |
|  | Rosenberg Self-Esteem Scale (RSES) | (Shu et al., 2019) |
|  | Beck Depression Inventory II | (Levinson et al., 2021) |
|  | Frost Multidimensional Perfectionism Scale (FMPS) | (Levinson et al., 2021) |
|  | | |
| Problematic Internet Use | PIU-SF-6 Problematic Internet Use Questionnaire | (Gansner et al., 2020) |
|  | PHQ-8 | (Gansner et al., 2020) |
|  | GAD-7 | (Gansner et al., 2020) |
|  | | |
| Conduct Problem | Diagnostic Interview Schedule for Children, Adolescents and Parents (DISCAP) | (Dadds et al., 2019) |
|  | Conners' Parent Rating Scale – Revised (CPRS-R) | (Dadds et al., 2019) |
|  | Strengths and Difficulties Questionnaire (SDQ) | (Dadds et al., 2019) |
|  | Brief Symptom Inventory (BSI) | (Dadds et al., 2019) |
|  | Scale to Assess the Therapeutic Relationship in Community Mental Health Care (STAR) | (Dadds et al., 2019) |
